# Hydrogen Peroxide, Povidone-Iodine and Chlorhexidine Fail to Eradicate *Staphylococcus aureus* Biofilm from Infected Implant Materials

**DOI:** 10.3390/life13061230

**Published:** 2023-05-23

**Authors:** Dana M. Parker, John A. Koch, Charles G. Gish, Kimberly M. Brothers, William Li, Jessica Gilbertie, Sarah E. Rowe, Brian P. Conlon, Venkata K. C. Byrapogu, Kenneth L. Urish

**Affiliations:** 1 Arthritis and Arthroplasty Design Lab, Department of Orthopaedic Surgery, University of Pittsburgh, Pittsburgh, PA 15261, USAbrothersk710@gmail.com (K.M.B.); liwt@upmc.edu (W.L.);; 2 Center for One Health Research, Edward Via College of Osteopathic Medicine, Blacksburg, VA 24060, USA; 3 Department of Microbiology and Immunology, University of North Carolina at Chapel Hill, Chapel Hill, NC 27599, USA; sarahrowe83@gmail.com (S.E.R.); brian_conlon@med.unc.edu (B.P.C.); 4 Marsico Lung Institute, University of North Carolina at Chapel Hill, Chapel Hill, NC 27599, USA; 5 Department of Bioengineering, University of Pittsburgh, Pittsburgh, PA 15261, USA

**Keywords:** antibiotic tolerance, biofilm, periprosthetic joint infection, oxidative stress, surgical infection

## Abstract

Hydrogen peroxide, povidone-iodine, and chlorhexidine are antiseptics that are commonly added to irrigants to either prevent or treat infection. There are little clinical data available that demonstrate efficacy of adding antiseptics to irrigants in the treatment of periprosthetic joint infection after biofilm establishment. The objective of the study was to assess the bactericidal activity of the antiseptics on *S. aureus* planktonic and biofilm. For planktonic irrigation, *S. aureus* was exposed to different concentrations of antiseptics. *S. aureus* biofilm was developed by submerging a Kirschner wire into normalized bacteria and allowing it to grow for forty-eight hours. The Kirschner wire was then treated with irrigation solutions and plated for CFU analysis. Hydrogen peroxide, povidone-iodine, and chlorhexidine were bactericidal against planktonic bacteria with over a 3 log reduction (*p* < 0.0001). Unlike cefazolin, the antiseptics were not bactericidal (less than 3 log reduction) against biofilm bacteria but did have a statistical reduction in biofilm as compared to the initial time point (*p* < 0.0001). As compared to cefazolin treatment alone, the addition of hydrogen peroxide or povidone-iodine to cefazolin treatment only additionally reduced the biofilm burden by less than 1 log. The antiseptics demonstrated bactericidal properties with planktonic *S. aureus*; however, when used to irrigate *S. aureus* biofilms, these antiseptics were unable to decrease biofilm mass below a 3 log reduction, suggesting that *S. aureus* biofilm has a tolerance to antiseptics. This information should be considered when considering antibiotic tolerance in established *S. aureus* biofilm treatment.

## 1. Introduction

Periprosthetic joint infection (PJI) is a major complication following total knee and hip arthroplasty with high morbidity and mortality [1,2,3]. For acute PJI and chronic PJI, irrigation and debridement are a critical portion of the procedure to minimize the recurrence of infection. Given the high failure rate [4], a variety of approaches to wound irrigation have been explored in an attempt to optimize irrigation [5,6]. In arthroplasty, hydrogen peroxide, povidone-iodine, and chlorhexidine irrigation solutions have been used in the prevention and treatment of PJI due to their increased bactericidal properties [7,8]. Hydrogen peroxide works by producing free hydroxyl radicals, causing oxidative damage [9]. Povidone-iodine releases free iodine, inducing degradation of important bacterial enzymes required for functioning [10]. Chlorhexidine works by causing coagulation around the bacteria, causing it to precipitate out, which ultimately prevents bacteria adhering to one another [11].

*Staphylococcus aureus* is the major pathogen responsible for PJI and can exist as two distinct phenotypes: a planktonic or biofilm phenotype [12]. Previous research demonstrated that irrigation with antiseptics can be used prophylactically to prevent acute PJI caused by planktonic bacteria; however, there is little research on whether irrigation with antiseptics can be used to treat PJI caused by established biofilms. In acute and chronic PJI, bacteria exist in the biofilm phenotype, especially on surgical implant materials and in synovial fluid [13,14]. When compared to their planktonic counterparts, biofilms are extremely tolerant to antimicrobials, making treatment challenging [15,16]. This tolerance is related to the reduced metabolic activity of the bacteria within the biofilm [17].

There has been minimal work investigating the ability of antiseptics to remove biofilm from implant materials. The objective of this study was to determine the utility of antiseptics alone to remove biofilm in an in vitro model of PJI. Given that reactive oxygen species can induce a decreased metabolic state [18], it was hypothesized that *S. aureus* biofilms would have an increased tolerance to antiseptics as compared to planktonic bacteria. In addition, combination treatment with antiseptics and a commonly used antibiotic, cefazolin, would have minimal advantage over antibiotics alone.

## 2. Materials and Methods

### 2.1. Bacterial Strains and Growth Conditions

The *S. aureus* SH1000 strain was inoculated from frozen stocks into 4 mL Tryptic Soy Broth (TSB, BD and Company, Franklin Lakes, NJ, USA) in a 15 mL conical tube at 37 °C with shaking at 250 rpm. Approximately 18 h later, cultures were diluted in PBS (Gibco, Billings, MT, USA) to the optical density OD_625_ using an Infinite M200 Spectrophotometer (Tecan, Männedorf, Switzerland) and normalized in TSB to a final concentration of 0.5 × 10^6^ colony forming unit (CFU)/mL using a 0.5 McFarland standard (Hardy Diagnostics, Santa Maria, CA, USA and Springboro, OH, USA). Conditions were based off Clinical & Laboratory Standards Institute (CLSI reference).

### 2.2. Planktonic Irrigation

In a 1.5 mL Eppendorf tube containing 0.5 × 10^6^ CFU/mL *S. aureus* SH1000 and 180 µL irrigation solution, bacteria were exposed to different antiseptics. The antiseptics that were used are as follows: chlorhexidine was assessed at 0.05% (Covetrus, Hastings, NE, USA); povidone-iodine (Purdue Products, Stamford, CT, USA) was assessed at 0.3%; and hydrogen peroxide (Equate, Bentonville, AR, USA) was assessed at 1.5%. All antiseptics, drugs and solutions were stored according to the directions provided by the manufacturers. The bacterial suspensions were exposed for 15 min to the antiseptics. The solutions were then diluted 1:100 in PBS to terminate the antiseptic activity. Next, each Eppendorf was serially diluted 10-fold and spot plated onto TSA II blood agar plates (Thermo Fisher Scientific, Waltham, MA, USA). After 24 h of incubation at 37 °C, the bacterial burden in CFU/mL was calculated for each antiseptic. Each experiment was completed in triplicate for each antiseptic and each concentration on three different days. To serve as a control, tubes with no bacteria and just antiseptics were plated to ensure no contamination occurred within the antiseptics.

### 2.3. Biofilm Irrigation

12” × 0.035” Kirschner wires (K wires) (Sklar, West Chester, PA, USA) were cut into 1 cm segments and autoclaved in sterile pouches before use. In a 48-well plate, a Kirschner wire was submerged into 1 mL of TSB containing 0.5 × 10^6^ CFU/mL *S. aureus* SH1000. To allow for biofilm maturation, the 48-well plates were cultured for 48 h at 37 °C. After the first 24 h of growth, the wires were removed and placed into fresh TSB to remove any planktonic/non-adherent cells. Forty-eight hours after initial plating, the K wires were removed from the media and washed with 2 mL of PBS to remove any remaining planktonic/non-adherent bacteria. Directly after washing, the K wires were placed into 1 mL of each antiseptic solution at the same concentrations as described in the planktonic irrigation above and exposed for 24 h. After treatment, the K wires were washed with 2 mL of PBS to remove the irrigation solution and placed into a 1.5 mL Eppendorf tube (Thermo Fisher Scientific, Waltham, MA, USA) containing 1 mL of PBS containing 1% Tween 20 (Sigma-Aldrich, St. Louis, MO, USA). The Eppendorf tube containing the K wire was sonicated for 30 min to dislodge any adhered biofilm. After sonication, the sonicate was serially diluted 10-fold and spot plated onto TSA II blood agar plates. After 24 h of incubation at 37 °C, colonies were counted, and the CFU/mL was calculated. Each experiment was completed in triplicate for each antiseptic and each concentration on three different days. To serve as a positive control, a non-treated wire was also diluted and used as the 0 min time point.

### 2.4. Antimicrobial Tolerance

K wires were infected with 0.5 × 10^6^ CFU/mL of *S. aureus* SH1000 as above in the biofilm irrigation methods. After 48 h of growth at 37 °C, wires were placed in each irrigation solution at the lowest concentration for 2.5 min. After exposure, the K wires were washed with PBS and placed into 1 mL of a 2.5 µg/mL cefazolin/TSB dilution for 24 h. At the 24 h time point, the K wires were washed with 2 mL PBS, sonicated for 30 min in a 1.5 mL Eppendorf tube containing 1 mL PBS, serially diluted 10-fold, and spot plated. CFU/mL was calculated after 24 h of incubation at 37 °C. Non-antiseptic treated K wires were also treated with 2.5 µg/mL of cefazolin for 24 h and served as a control. The experiment was completed in triplicate for each antiseptic at each time point. To serve as an untreated control, non-irrigated wires were evaluated at the 0 h control time point.

### 2.5. Statistical Analysis

GraphPad Prism 9.0 was used to perform all graphical and statistical analysis. Statistical analyses were performed using one-way ANOVA with Tukey’s post hoc analysis. Significance was determined at *p* < 0.05. All experiments were repeated in triplicate.

## 3. Results

### 3.1. Antiseptics Are Bactericidal against S. aureus Planktonic Bacteria

**Figure 1:**
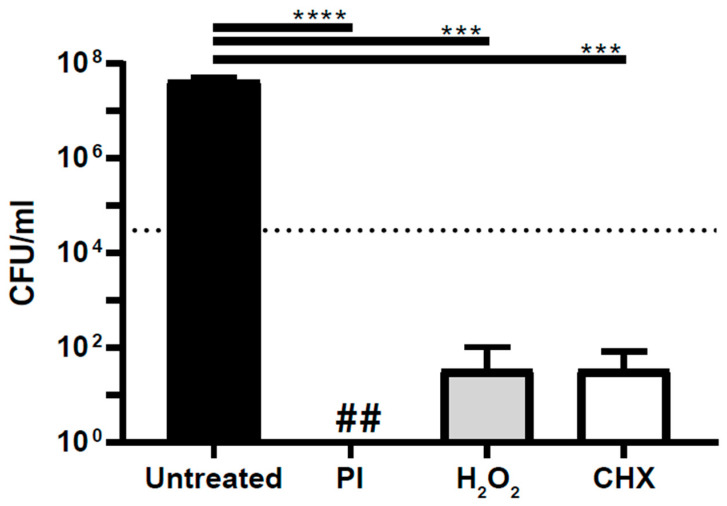
Antiseptics are bactericidal against *S. aureus* planktonic bacteria. The amount of *S. aureus* SH1000 in suspension after antiseptic treatment was quantified and compared. PI—povidone-iodine at 0.3%; CHX—chlorhexidine at 0.05%; and H_2_O_2_—hydrogen peroxide at 1.5%; . . . . . = 3 log reduction; ## = undetectable amount; *** *p* < 0.001; **** = *p* < 0.0001.

To determine if the antiseptics are bactericidal against *S. aureus* planktonic bacteria, an irrigation assay was used to quantify the amount of *S. aureus* planktonic bacteria remaining after treatment with each antiseptic. Clinically relevant concentrations of each antiseptic were chosen based on the literature [5,19]. Povidone-iodine, chlorhexidine, and hydrogen peroxide all significantly decreased planktonic bacterial load (*p* < 0.0001, *p* < 0.001, *p* < 0.001) (Figure 1). Specifically, povidone-iodine, hydrogen peroxide, and chlorhexidine reduced the bacterial burden by a 7.6, 6.1, and 6.1 log reduction, respectively. In comparison to untreated bacteria, povidone-iodine completely eradicated *S. aureus* planktonic bacteria, while hydrogen peroxide and chlorhexidine significantly reduced *S. aureus*.

### 3.2. Antiseptics Are Not Bactericidal against S. aureus Biofilms

**Figure 2:**
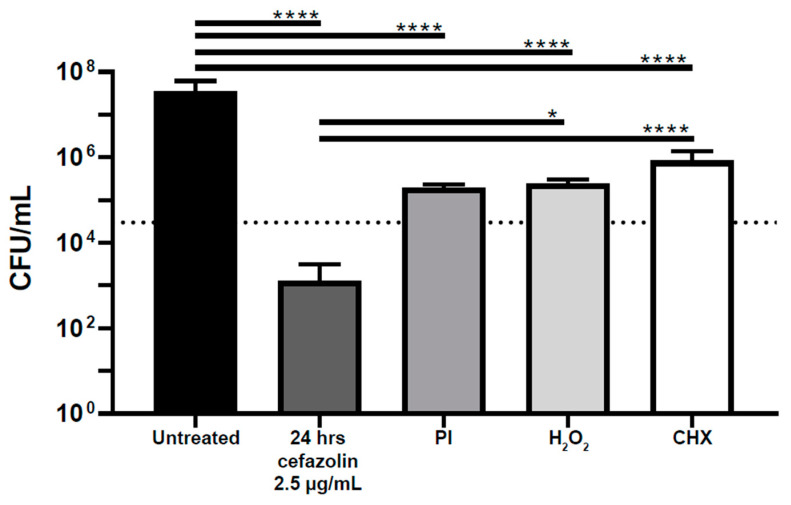
Antiseptics are not bactericidal against *S. aureus* biofilm. The amount of *S. aureus* biofilm after treatment was quantified and compared. PI—povidone-iodine at 0.3%; CHX—chlorhexidine at 0.05%; and H_2_O_2_—hydrogen peroxide at 1.5%; . . . . . = 3 log reduction; * = *p* < 0.01; **** = *p* < 0.0001.

Next, a biofilm assay was used to determine if antiseptics were equally effective against *S. aureus* biofilms as they demonstrated with planktonic bacteria. The same dosing and exposure times were used as in the planktonic assay. Povidone-iodine, hydrogen peroxide, and chlorhexidine were not observed to have bactericidal properties against biofilms as defined by a 3 log reduction. Each antiseptic had a decrease in *S. aureus* biofilm bacterial burden in comparison to untreated biofilms by 2.2, 2.2, and 1.6 log, respectively (*p* < 0.0001) (Figure 2).

### 3.3. Antiseptics Are Not Synergistic with Cefazolin against S. aureus Biofilms

**Figure 3:**
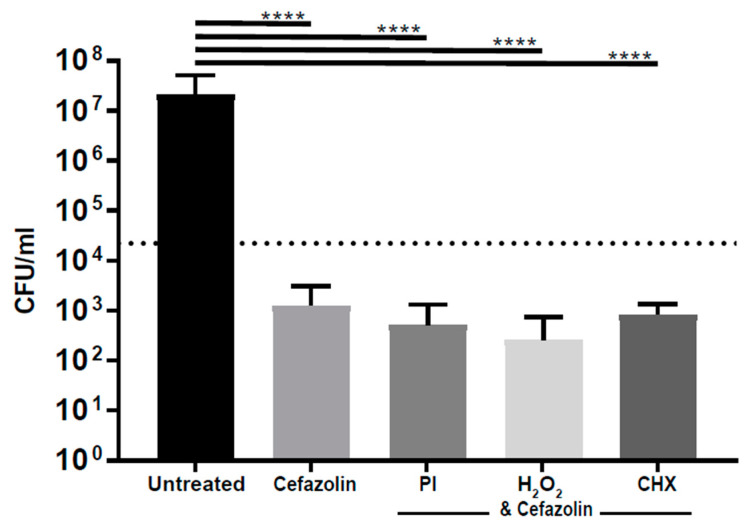
Antiseptics in combination with cefazolin reduces biofilm but does not display synergy. The amount of *S. aureus* present after treatment with cefazolin in combination with antiseptics was quantified and compared. CHX—chlorhexidine at 0.05%; PI—povidone-iodine at 0.3%; H_2_O_2_—hydrogen peroxide at 1.5%; and cefazolin = 2.5 μg/mL; . . . . . = 3 log reduction; **** *p* < 0.0001.

To further investigate the relationship between the antimicrobial tolerance of *S. aureus* biofilms and antiseptics, we treated *S. aureus* biofilms with a combination of antiseptics and cefazolin. The addition of cefazolin (10 × MIC) to povidone-iodine, hydrogen peroxide and chlorhexidine resulted in a 4.6, 4.9, and 4.5 log reduction (*p* < 0.0001), respectively, in comparison to the untreated group. Compared to cefazolin treatment alone, the combination of cefazolin with povidone-iodine, hydrogen peroxide or chlorhexidine resulted in a 0.40, 0.70, and 0.20 log reduction, respectively (Figure 3), and was not statistically significant (*p* > 0.99).

## 4. Discussion

The utility of irrigation solutions to treat PJI remains undefined. There is clinical evidence supporting the use of oxidative antiseptics, including povidone-iodine, hydrogen peroxide and chlorhexidine as prophylactic irrigation solutions to prevent PJI when bacteria are in the planktonic state [19,20,21,22,23,24]. There is limited data available on if these oxidative antiseptics irrigation solutions improve the treatment of PJI when bacteria are in the biofilm state. Previous work has demonstrated that oxidative stress can induce an antibiotic-tolerant biofilm [18], and this suggests that antiseptics have the potential to decrease the efficacy of antibiotics in the treatment of PJI. The objective of this study was to determine the utility of antiseptics in removing biofilm in an in vitro model of PJI. Based on the data, *S. aureus* biofilm had a high tolerance to antiseptics as compared to planktonic bacteria. Synergism was not observed between cefazolin and the antiseptics used.

Standard antiseptic irrigation solutions used intraoperatively were bactericidal against planktonic bacteria in the in vitro experiment preformed. Povidone-iodine, hydrogen peroxide, and chlorhexidine had more than a 3 log reduction in planktonic *S. aureus*, which is considered bactericidal. This is consistent with multiple previous in vitro studies demonstrating similar results [25,26,27,28]. This supports the utility of adding antiseptic solutions to intraoperative irrigation solutions to prevent surgical site infection and PJI. These in vitro findings are supported by a series of clinical studies that have observed a possible decrease rate in the incidence of PJI by using a variety of different antiseptic irrigation solutions [19,20,21,22,23,24]. There is caution against using antiseptics based on possible cytotoxicity issues, especially with the use of chlorhexidine [28,29].

Povidone-iodine, hydrogen peroxide, and chlorhexidine decreased biofilm burden, but were not bactericidal to *S. aureus* biofilm. This is supported by other in vitro studies [18,30]. There is a lack of clinical evidence supporting the antimicrobial efficacy of antiseptics in treating PJI. The logic to use antiseptics to treat PJI is largely based on extending results from clinical studies demonstrating these irrigation solutions help prevent PJI. These studies have limited validity once PJI is established [19,20,21,22,23,24]. A retrospective study of over 2800 revision arthroplasties demonstrated that the use of intraoperative povidone-iodine had no effect on PJI treatment success during revision cases [31]. We observed that like antibiotics, antiseptics were not bactericidal once *S. aureus* was in a biofilm state. Our data should not be interpreted against the use of these irrigation solutions. A reduction in biofilm was observed with the antiseptic solutions supporting their clinical use; however, the reduction was not bactericidal. This does demonstrate the challenge in treating biofilm-associated infections and the need for independent irrigation solutions that have the potential to be bactericidal against an established biofilm.

Povidone-iodine, hydrogen peroxide, and chlorhexidine in combination with cefazolin did not display synergy against *S. aureus* biofilm. There were no statistical differences in biofilm antimicrobial efficacy between cefazolin and cefazolin in combination with the antiseptics. This adds to growing evidence that povidone-iodine, hydrogen peroxide, and chlorhexidine with cefazolin could potentially induce antibiotic tolerance. During the host immune response, hydrogen peroxide operates via an oxidative-stress-response pathway. Oxidative-stress-rich environments, similar to those found in *S. aureus* PJI, can induce persister cell formation that leads to increased antimicrobial tolerance, which can result in higher likelihood of chronic infection [18,32,33]. Reactive oxygen species, such as hydrogen peroxide, promote increased antibiotic tolerance in *S. aureus* [18]. This is likely because *S. aureus* biofilm enter a metabolically inactive state and form persister cells, which conveys increased tolerance to antimicrobials [12,34,35].

There are several limitations to our study. First, this was conducted in vitro and thus does not replicate the physiological environment of biofilms in vivo. Typically, in vitro models result in a best-case scenario and further validation of these results in PJI animal models is warranted. Second, a supratherapeutic dose of cefazolin for a prolonged time period was used in combination with antiseptics. This does not necessarily replicate a clinical environment, where concentrations of antibiotic are more limited with variations between peaks and troughs, suggesting a best-case scenario for our results.

## 5. Conclusions

Our study demonstrates, in an in vitro model, that antiseptics such as povidone-iodine, hydrogen peroxide and chlorhexidine can significantly decrease the bacterial load of planktonic *S. aureus* but fail to significantly eradicate *S. aureus* biofilms. This supports their use to help prevent PJI in primary hip, knee, and shoulder arthroplasty cases. In treating PJI, our data supports their use but highlights the limitations against biofilms that are antibiotic- and antiseptic-tolerant. The addition of cefazolin to povidone-iodine, hydrogen peroxide, and chlorhexidine was not significantly beneficial. Further studies involving in vivo experiments in animal models are needed to elucidate these effects in a physiologic setting. This would provide additional support or disprove the results in this study. Furthermore, additional studies involving other organisms common to PJI would serve as good comparison data and possibly lead to a better understanding of biofilm characteristics.

## Data Availability

The data presented in this study are available upon request from the corresponding author. The data are not publicly available due to ongoing research.
